# An innovative educational program for addressing health disparities in translational cancer research

**DOI:** 10.1017/cts.2020.555

**Published:** 2020-11-05

**Authors:** Carla E. Oldham, M. J. Gathings, Gayathri R. Devi, Steven R. Patierno, Kevin P. Williams, Holly J. Hough, Nadine J. Barrett

**Affiliations:** 1 Biomanufacturing Research Institute Technology Enterprise (BRITE) and Department of Pharmaceutical Sciences, North Carolina Central University, Durham, NC, USA; 2 ETR Services, LLC, Durham, NC, USA; 3 Division of Surgical Sciences, Department of Surgery, Duke University School of Medicine, Durham, NC, USA; 4 Department of Pathology, Duke University School of Medicine, Durham, NC, USA; 5 Duke Cancer Institute, Duke University Medical Center, Durham, NC, USA; 6 Division of Medical Oncology, Department of Medicine, Duke University School of Medicine, Durham, NC, USA; 7 Office of Clinical Research, Duke University School of Medicine, Durham, NC, USA; 8 Clinical and Translational Science Institute, Duke University School of Medicine, Durham, NC, USA; 9 Department of Family Medicine and Community Health, Duke University School of Medicine, Durham, NC, USA.

**Keywords:** Cancer, translational research, pipeline, education, training, community engagement, disparities, clinical trials, diversity

## Abstract

North Carolina Central University (NCCU) and Duke Cancer Institute implemented an NCI-funded Translational Cancer Disparities Research Partnership to enhance translational cancer research, increase the pool of underrepresented racial and ethnic group (UREG) researchers in the translational and clinical research workforce, and equip UREG trainees with skills to increase diversity in clinical trials. The Cancer Research Education Program (C-REP) provided training for UREG graduate students and postdoctoral fellows at Duke and NCCU. An innovative component of C-REP is the Translational Immersion Experience (TIE), which enabled Scholars to gain knowledge across eight domains of clinical and translational research (clinical trials operations, data monitoring, regulatory affairs, UREG accrual, biobanking, community engagement, community outreach, and high-throughput drug screening). Program-specific evaluative metrics were created for three broad domains (clinical operations, basic science/lab research, and population-based science) and eight TIE domains. Two cohorts (*n* = 13) completed pre- and post-surveys to determine program impact and identify recommendations for program improvement. Scholars reported statistically significant gains in knowledge across three broad domains of biomedical research and seven distinct areas within TIE. Training in translational research incorporating immersions in clinical trials operation, biobanking, drug development, and community engagement adds value to career development of UREG researchers.

## Introduction

Two key and interrelated challenges to cancer disparities research include the lack of investigators from underrepresented racial and ethnic groups (UREGs) contributing to cancer research and the underrepresentation of UREG participants in clinical trials [1]. Despite mandates to increase diversity in clinical research participation, there remains little or no change in the paucity of minorities involved in research [2–4]. Since the passage of the 1993 National Institutes of Health (NIH) Revitalization Act, which required all clinical research funded by the federal government to include women and minorities, less than 2% of over 10,000 cancer studies have included enough minorities to meet NIH’s criteria [5, 6].

Inadequate participation of diverse UREGs in clinical research and trials compromises generalizability of research findings and fosters biased reporting of both therapeutic and adverse effects that may differ by race, resulting in insufficient data to assess the efficacy or safety of new treatments and drugs [7]. This disparity also limits the discovery, development and dissemination of novel, and perhaps population-specific prevention and intervention strategies that could mitigate, if not eliminate, cancer disparities in traditionally underserved racial and ethnic communities.

Similarly, a significant disparity exists across the academic pipeline where UREG students are less likely to complete undergraduate or graduate degrees in the biological sciences. When they do matriculate, underrepresented students are less likely to pursue a path towards research independence [8]. For example, only 50% of UREG students who declare a STEM (Science, Technology, Engineering, and Mathematics) major receive an undergraduate STEM degree when compared to an 88% retention rate among majority students [9]. Although the number of doctoral degrees awarded to UREGs is increasing, the rates have not kept pace with the changing demographics in the general population. UREG faculty representation follows a similar upward trend in some areas of the country, but at present only 4% of full-time faculty in medical schools are from underrepresented groups [10].

These factors highlight two urgent and compelling needs: (a) to provide vital translational cancer disparities research training opportunities for UREG trainees to diversify the research workforce and (b) to arm scholars with comprehensive training emphasizing clinical trials operational infrastructure, evidence-based strategies to increase diverse participation in biomedical research – particularly among UREGs, and link trainees to community engagement and outreach.

## Methods

The Cancer Research Education Program (C-REP), a core and required component of an NCI-funded P20 grant (NCCU-Duke Translational Cancer Disparities Research Partnership), was developed as an innovative educational collaboration between a Comprehensive Cancer Center, the Duke Cancer Institute (DCI), and a historically black university, North Carolina Central University (NCCU). C-REP addressed three critical needs in translational cancer disparities research and training: (a) increasing diversity in the cancer disparities research training pipeline and workforce, (b) training the next generation of translational scientists in clinical trials operations, processes, and strategies to increase UREG accrual, and (c) increasing researchers’ awareness, knowledge and skills in community outreach and engagement as a key component of the translational research spectrum.

**Fig. 1.**
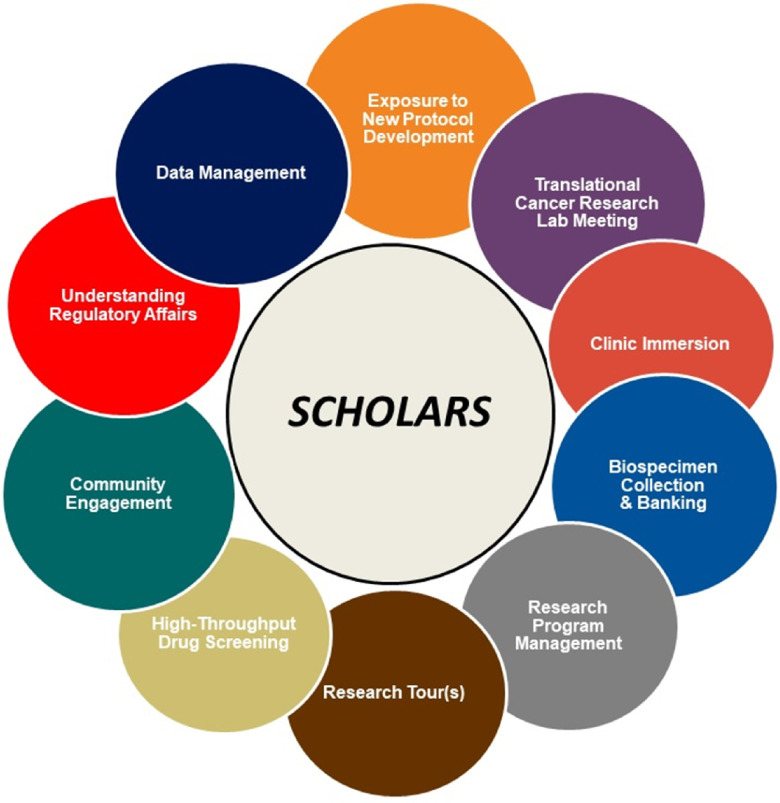
Components of the Translational Immersion Experience (TIE).

This 2-year program included traditional training and educational activities that exposed graduate and postdoctoral scholars to cancer disparities translational research, professional development opportunities (e.g., grant writing, career development workshops), and cross-institutional mentoring. Trainees also attended two mentorship workshops that focused on improving communication, aligning expectations, and diversity and inclusion. Providing mentorship training around bias, inequities, and strategies to mitigate their effects was an innovative component of the program designed to increase retention among UREG trainees in the academic pipeline. Discussions of case studies provided opportunities for trainees to improve their skills in recognizing biases and prejudices, working across differences (i.e., age, race, ethnicity, gender, class, religion, and sexual orientation), and addressing issues of equity and inclusion within professional settings. Although C-REP was a required component of the P20 grant, the Translational Immersion Experience (TIE), a program within C-REP, was designed by the grant team to provide a series of interactive, experiential, and educational opportunities that enabled scholars to expand their skills and knowledge around the structure, processes, and roles within clinical trial operations, high-throughput drug screening, strategies to address lack of UREG participation in clinical research, and the importance and practice of community engagement and outreach within the translational research spectrum (Fig. 1).

### Translational Immersion Experience

#### C-REP Scholars

Trainees who participated in C-REP included UREG doctoral students and postdoctoral fellows from NCCU and Duke. The program was structured around two cohorts of scholars. We conducted outreach campaigns using diverse platforms, including listservs, programs, workshops, and departments geared towards UREG graduate students and postdoctoral fellows across both institutions. Prospective candidates submitted applications outlining career and research goals, letters of recommendation, and a curriculum vitae. A review committee assessed applications based on each candidate’s career and research goals and their alignment with the program.

The TIE was comprised of two summer sessions. Each cohort participated in the introductory TIE during the summer of their first program year. During the first summer, eight TIE sessions occurred every 2 weeks lasting 2 hours each. Facilitators included staff and investigators from both NCCU and Duke who were part of the larger P20 partnership. Scholars experienced a deeper dive into particular domains during the second summer session. In this communication, we describe the introductory TIE program (i.e., a sequence of immersive workshops that trainees attend during their first year) and evaluative findings for both cohorts.

#### TIE Curriculum

Scholars engaged in a series of educational sessions along the translational spectrum, including clinical trials operations, UREG accrual in clinical trials, and community engagement. Sessions were led by experts at NCCU, Duke, and industry leaders, such as representatives from contract research organizations and pharmaceutical companies, who were invited to share diverse perspectives from outside of the two institutions. The TIE sessions began with an **Introductory Seminar** that consisted of an in-depth analysis of an active clinical trial through a disparities lens, which provided the framework and context of translational cancer disparities research across the spectrum. Scholars attended a **Translational Research Lab Meeting** where protocols were developed and discussed and all members of the research team attended and contributed, including the Principal Investigator(s). The **Regulatory** and **Data Monitoring** sessions allowed scholars to gain a deeper understanding of protocol development, regulatory requirements, and key operations around data collection, storage, and analysis.

Scholars also toured the **Duke BioRepository and Precision Pathology Center** and the **Duke Early Phase Clinical Research Unit**. This full experiential learning session started in the biobank where scholars learned what the biobank does and the methodology for storing tissue. Scholars also had an opportunity to experience preparing tissue for the biorepository. Scholars then toured the Duke Early Phase Research Clinic where they learned more about early phase studies and the resources required to operate the clinic and accelerate the availability of therapies, diagnostics, and medical devices to the broader population. Scholars attended a session on **Clinical Trial Operations**, which provided orientation to the clinical aspects of oncology research and included shadowing a variety of clinical research team members, including the physician, research nurse, and/or the clinical research coordinator, to gain an understanding of how clinical trials are operationalized. The **High-Throughput Screening** experience was a 2-day training at the Biomanufacturing Research Institute and Technology Enterprise (BRITE) Institute at NCCU where scholars learned about automation and liquid handling, detection devices, and data processing and software that has the ability to perform thousands of pharmacological experiments. Scholars received an overview and learned assay design for the purpose of drug discovery, which included assay development, optimization and validation, screening, and data analysis.

The **Community Engagement and Diversity in Clinical Trial Session** was both an immersion and educational session in which scholars learned the principles of community and patient engagement, strategies to address these issues along the translational spectrum, and gained a deeper understanding of disparities in cancer and the social determinants of health. Scholars learned barriers to diversity in clinical trials as well as novel and proactive strategies to address these barriers in their own work. Scholars also participated in two community outreach programs each year: the Men’s Health Initiative (sponsored by the Duke Cancer Institute and Lincoln Community Health Center) and Women’s Health Awareness Day (developed and funded by the National Institute of Environmental Health Sciences, hosted by North Carolina Central University). Both events provided no-cost screenings and patient navigation services to attendees. Lastly, a **Career Panel and Networking** session was held where scholars were introduced to diverse careers in clinical research settings, including academic institutions, pharmaceutical companies, and contract research organizations. Collectively, this novel training program was designed to ensure scholars were immersed in key areas of clinical research operations and community engagement, and have a more comprehensive view of translational cancer disparities research and the impact they can have as research scientists in their field and the community.

## Results

A total of 14 C-REP scholars were selected: 7 were Black/African-American, 4 were Latinx/Hispanic, and 3 were Mixed Race/Other. Twelve were doctoral students and two were postdoctoral fellows (one at each institution). All C-REP Scholars (seven from NCCU and seven from Duke) were basic scientists.

To evaluate the effectiveness of the TIE, program-specific evaluative metrics were created for three broad domains of biomedical science and all eight immersion topics. Two cohorts of C-REP Scholars completed an online pre-post survey during Summer 2018 and Summer 2019, respectively (*n* = 13). In the results that follow, continuous data are presented as mean ± standard deviation (SD) in conjunction with results from paired sample *t* tests. Paired sample *t* tests indicate whether pre- and post-assessment scores were statistically significant, thus reflecting meaningful changes in self-reported knowledge and confidence. Significance was established at the .05 level.

As Table 1 shows, trainees reported increased knowledge of and confidence in their abilities to perform within the three broad domains of science examined. C-REP Scholars reported the largest gains in knowledge of clinical operations (from 3.08 ± 1.320 to 5.08 ± 1.256) and population-based research (from 3.15 ± 1.519 to 4.62 ± .870). Paired *t* tests show statistically significant increases in scholars’ self-reported knowledge of clinical operations (*P* < .001), knowledge of basic science and lab research (*P* < .05), and knowledge of population-based studies (*P* < .001). Two of three confidence metrics were statistically significant, including confidence in abilities to perform clinical operations (from 3.62 ± 2.063 to 4.69 ± 1.437, *P* < .05) and basic science/lab research (from 6.23 ± .725 to 6.54 ± .519, *P* < .05).

**Table 1.** Comparison of self-reported knowledge^†^ and confidence^‡^ on pre- and post-immersion assessments for two cohorts completing a translational immersion experience through Duke and NCCU’s cancer research and training program^*^

	Pre	Post	
	Mean ± SD	Mean ± SD	*P*-value
**Clinical operations**			
Knowledge of	3.08 ± 1.320	5.08 ± 1.256	.001
Confidence in abilities	3.62 ± 2.063	4.69 ± 1.437	.042
**Basic science/lab research**			
Knowledge of	5.83 ± .835	6.33 ± .778	.026
Confidence in abilities	6.23 ± .725	6.54 ± .519	.040
**Population-based research**			
Knowledge of	3.15 ± 1.519	4.62 ± .870	.001
Confidence in abilities	4.15 ± 2.115	4.46 ± 1.050	.487

* SD indicates standard deviation. Likert scale items were measured on a seven-point scale ranging from (1) very poor to (7) excellent, unless otherwise noted.

† Item asked: “Using a scale from 1–7 where 1 indicates very poor and 7 means excellent, please rate your knowledge in the following areas.”

‡ Item asked: “On a scale of 1–7 where 1 is very poor and 7 is excellent, how would you rate your confidence in your abilities to perform.”

Table 2 details changes in trainees’ knowledge on topics covered throughout their immersion experience. Trainees reported increased knowledge in all eight areas with the largest gains in operating clinical trials in clinical environments (from 2.85 ± 1.864 to 5.31 ± 1.182) and UREG accrual (from 2.73 ± 1.272 to 5.00 ± 1.183). C-REP Scholars reported statistically significant increases in knowledge in seven of the eight session topics: high-throughput screening (*P* < .05), operating clinical trials in a clinical environment (*P* < .001), data monitoring (*P* < .05), regulatory operations (*P* < .001), UREG accrual (*P* < .001), bio-banking (*P* < .001), and community engagement (*P* < .05). At the end of their summer immersion experience, trainees ranked their preferences for a deeper immersion in Year 2: approximately 38% of trainees ranked clinical immersion as their first choice for deeper immersion with both regulatory affairs and high-throughput screening each selected by 15% of trainees.

**Table 2.** Comparison of self-reported knowledge^†^ on pre- and post-immersion assessments two cohorts completing a translational immersion experience through duke and NCCU’s cancer research and training program^*^

	Pre	Post	
	Mean ± SD	Mean ± SD	*P*-value
High throughput screening	3.54 ± 2.106	5.23 ± 1.739	.013
Clinical trials in clinical environment	2.85 ± 1.864	5.31 ± 1.182	.001
Data monitoring	3.46 ± 1.898	5.08 ± 1.115	.003
Regulatory operations	2.77 ± 1.739	4.85 ± 1.281	.000
UREG accrual	2.73 ± 1.272	5.00 ± 1.183	.001
Bio-banking	2.38 ± 1.609	4.46 ± 1.613	.001
Community outreach	4.92 ± 1.165	5.50 ± 1.000	.111
Community engagement	4.62 ± 1.193	5.38 ± .961	.026

* SD indicates standard deviation. Likert scale items were measured on a seven-point scale ranging from (1) very poor to (7) excellent, unless otherwise noted.

† Item asked: “Using a scale from 1–7 where 1 indicates very poor and 7 means excellent, please rate your knowledge in the following areas.”

Community outreach was an important facet of training, noting the importance of understanding barriers and facilitators to increasing diverse participation and the role of community engagement around research participation – an area most trainees do not receive training in as they go through the traditional curriculum. Interestingly, gains in knowledge of community engagement were statistically significant while reported knowledge gains in community outreach were not. Yet qualitative data collected through interviews and observations suggest outreach events provided a meaningful opportunity to engage the public. One participant noted the program’s impact on his/her future career aspirations: “*During my graduate studies, I didn*’*t do community outreach but it*’*s something I*’*m leaning towards now*. [TIE] *is a really good exposure to community outreach*.” Other trainees noted feelings of personal fulfillment in the wake of their participation in community outreach efforts, As one trainee elaborated, “*The Men*’*s Health Day Initiative was great to see, especially how many people we were able to improve health outcomes for…it meant a lot to give back to the community in that way.”* Another C-REP Scholar shared, “*As a graduate student from an underrepresented group, I have developed a deep appreciation for participating in events where I can share my time and training to serve my community. This particular experience has shaped my perspective on how much more impactful these types of initiatives can be for patients/individuals when they are able to talk and ask questions to someone who looks and talks like them*.”

Trainee placements after graduation provide further support for positive program impacts on career development. C-REP trainees to date have successfully transitioned to positions within clinical research: one trainee is in a postdoctoral fellowship with the federal government, two accepted positions with contract research organizations, one is continuing educational training in the Duke Medical Scientist Training Program, and one NCCU Scholar was hired as a postdoctoral research associate at the Duke Cancer Institute. The postdoctoral fellow at Duke recently won the 2020 AACR-Genentech Cancer Disparities Research Fellowship. The remaining scholars will be completing their doctoral programs in the next 1–2 years. Taken collectively, C-REP scholars gave 17 conference presentations, 2 grants were awarded, 3 articles were published or accepted for publication, and 3 manuscripts were under review.

## Discussion

Increased knowledge and exposure to contract research organizations, translational research, increasing diversity in clinical trials and community engagement were the principal goals in the program for Year 1. Through participation in the TIE, trainees built their knowledge of health disparities in translational cancer research, clinical operations, and community engagement, the latter being an area in which basic/bench scientists often have limited exposure or experience. Hands-on involvement in outreach events, such as the DCI’s Men’s Health Initiative, provided important opportunities for Scholars to experience firsthand the impact of their involvement in outreach efforts to vulnerable communities. The combination of training, hands-on experience in community outreach, and networking among established professionals has better prepared trainees to positively impact UREG accrual, diversity in the cancer disparities research workforce and pipeline, and engagement in community outreach as part of the translational research spectrum.

With current patterns of uneven representation of UREG scholars across the academic pipeline, there is a pressing need for training programs that center around the experiences of UREG scholars. C-REP provides an innovative model for preparing underrepresented researchers to enter the translational research workforce by (1) providing mentorship training with an explicit focus on navigating issues of diversity and inclusion; and (2) providing exposure to the different phases of clinical operations. The C-REP TIE provides trainees with novel and tangible experiences and skills that set them apart from other peer researchers in biological sciences and expand their professional opportunities after matriculation.

Preliminary results suggest that scholars were impacted in seven of the eight sessions within TIE. In the future, both cohorts will participate in a second rotation of TIE and gain more focused experience in one of eight areas. From this exposure, we predict that trainees will learn more about various aspects of clinical trial operations, gain more perspective about career options within clinical research, and ultimately determine viable career trajectories for themselves. As UREGs, C-REP Scholars will have a broader set of skills and knowledge in both clinical and translational cancer research and the importance of diversity in research participation and community engagement. These domains are not explored in the traditional training curriculum for basic science graduate students and postdocs and yet provide added value to the strengths each C-REP Scholar brings to the translational research workforce, further contributing to the elimination of cancer health disparities, and improving population and community health.

While our program is focused on increasing UREG representation in the clinical and translational research workforce, we also see that key elements of this training could be beneficial for non-UREG trainees in providing skills for community engagement, clinical trial operations, and strategies for improving diversity in clinical trials. Future program iterations may incorporate project-based assignments, such as team science in health disparities and/or integrate more hands-on experience around increasing diverse participation in clinical trials. Future evaluations of similar initiatives could employ additional objective measures of professional development and career performance as well as quasi-experimental methods that compare measures of diverse study recruitment for early career investigators who participated in C-REP with those who did not.

Overall, this program leverages significant strengths across both institutions, which includes (a) cutting-edge research that covers the translational spectrum, including strong community engagement programs and activities and a clinical trials platform at Duke and (b) NCCU’s Biomanufacturing Research Institute and Technology Institute (BRITE), offering hands-on education and training in key areas of drug development and biomanufacturing. Other resources that are important in adapting C-REP to other institutions are a strong network of mentors, an established network of biotechnology/contract research organizations, and dedicated staff time for implementation and evaluation. Together, these strengths are critical components that have led to a successful program and, to date, allowed Scholars to matriculate to the next level of their career in translational research.
